# Supplementary material for: Ginkgetin attenuates bone loss in OVX mice by inhibiting the NF-κB/IκBα signaling pathway
Source: PeerJ. 2024 Jul 10;12:e17722. doi: 10.7717/peerj.17722 (PMC11246017; doi:10.7717/peerj.17722)
Supplement: Supplemental Information 1 [file peerj-12-17722-s001.zip › wb.docx]

**Figure 1:** p-P65:


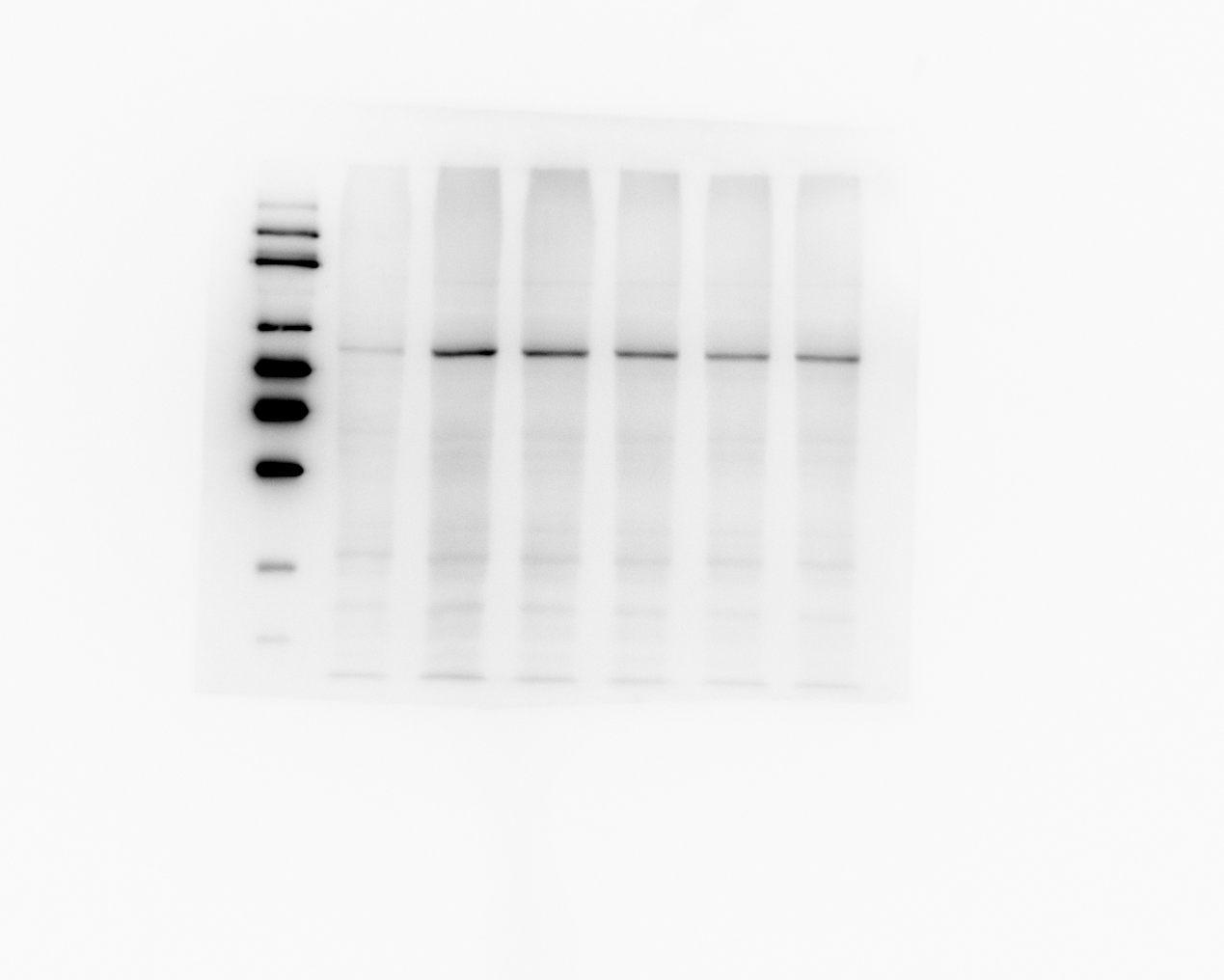


Groups (From left to right)：(1) Sham; (2) OVX; (3) OVX+ Ginkgetin (low); (4) OVX+ Ginkgetin (mid); (5) OVX+ Ginkgetin (high); (6) OVX+PGE.

**Figure 2:** P65:


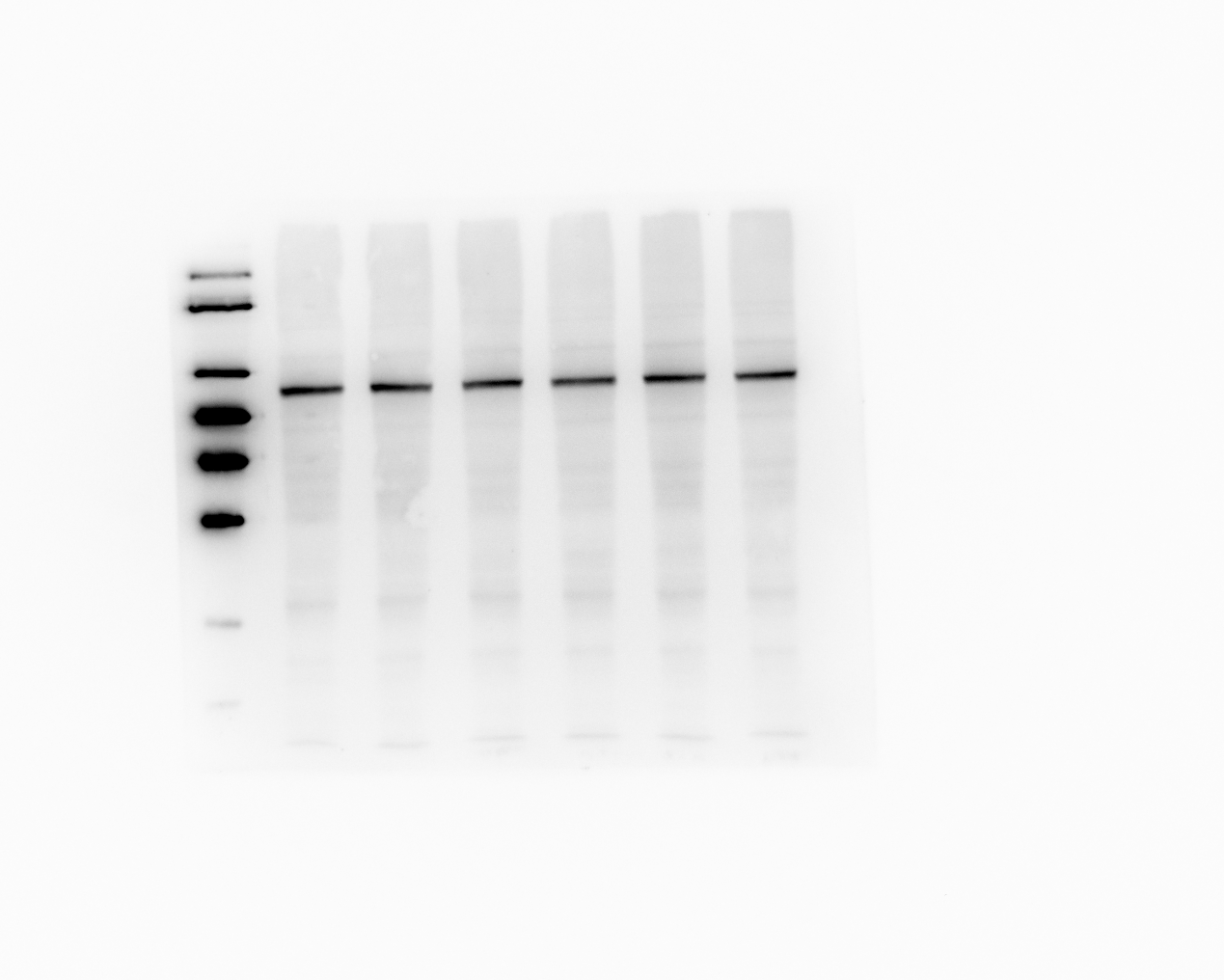


Groups (From left to right)：(1) Sham; (2) OVX; (3) OVX+ Ginkgetin (low); (4) OVX+ Ginkgetin (mid); (5) OVX+ Ginkgetin (high); (6) OVX+PGE.

**Figure 3:** IκBα


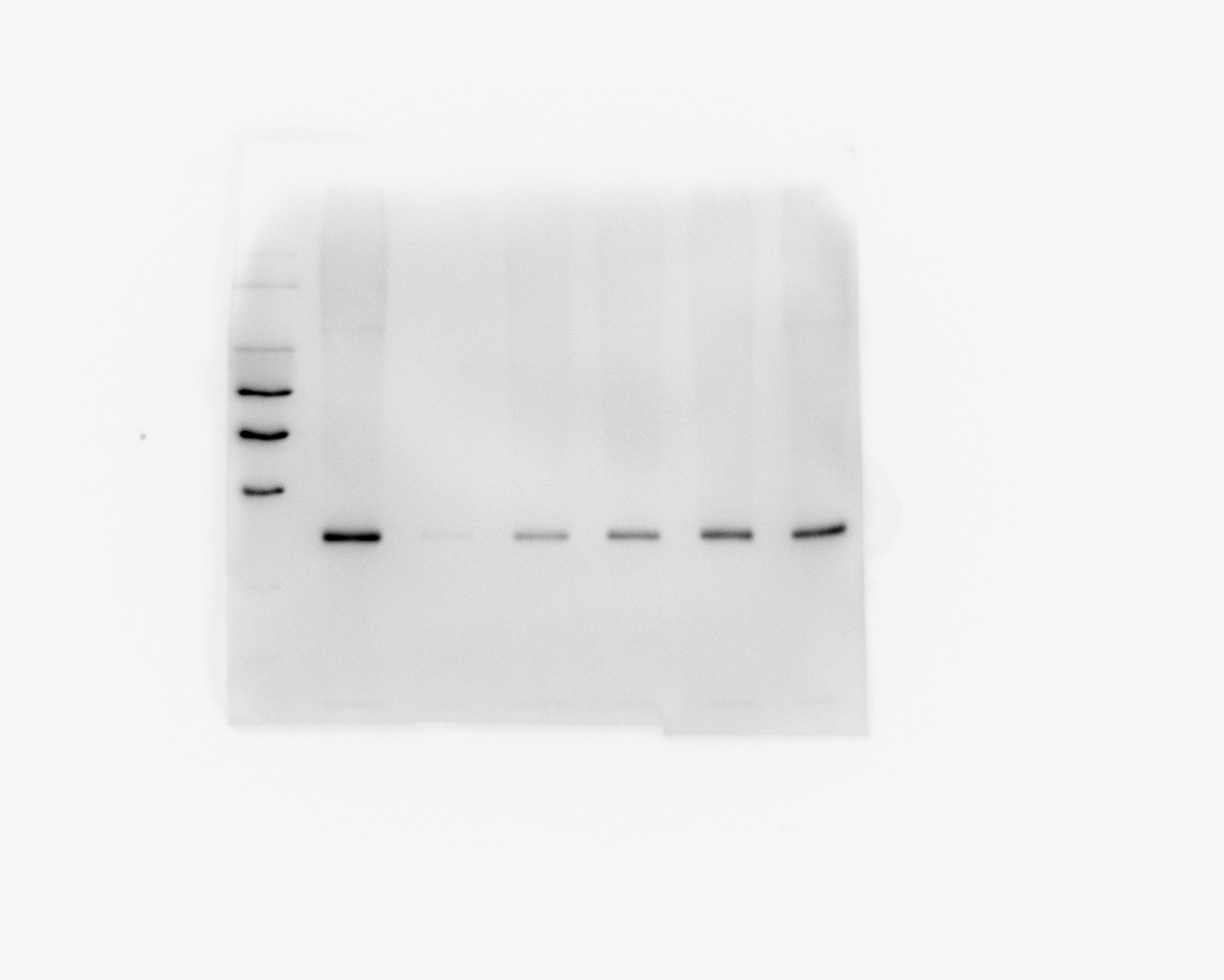


Groups (From left to right)：(1) Sham; (2) OVX; (3) OVX+ Ginkgetin (low); (4) OVX+ Ginkgetin (mid); (5) OVX+ Ginkgetin (high); (6) OVX+PGE.

**Figure 4:** GAPDH


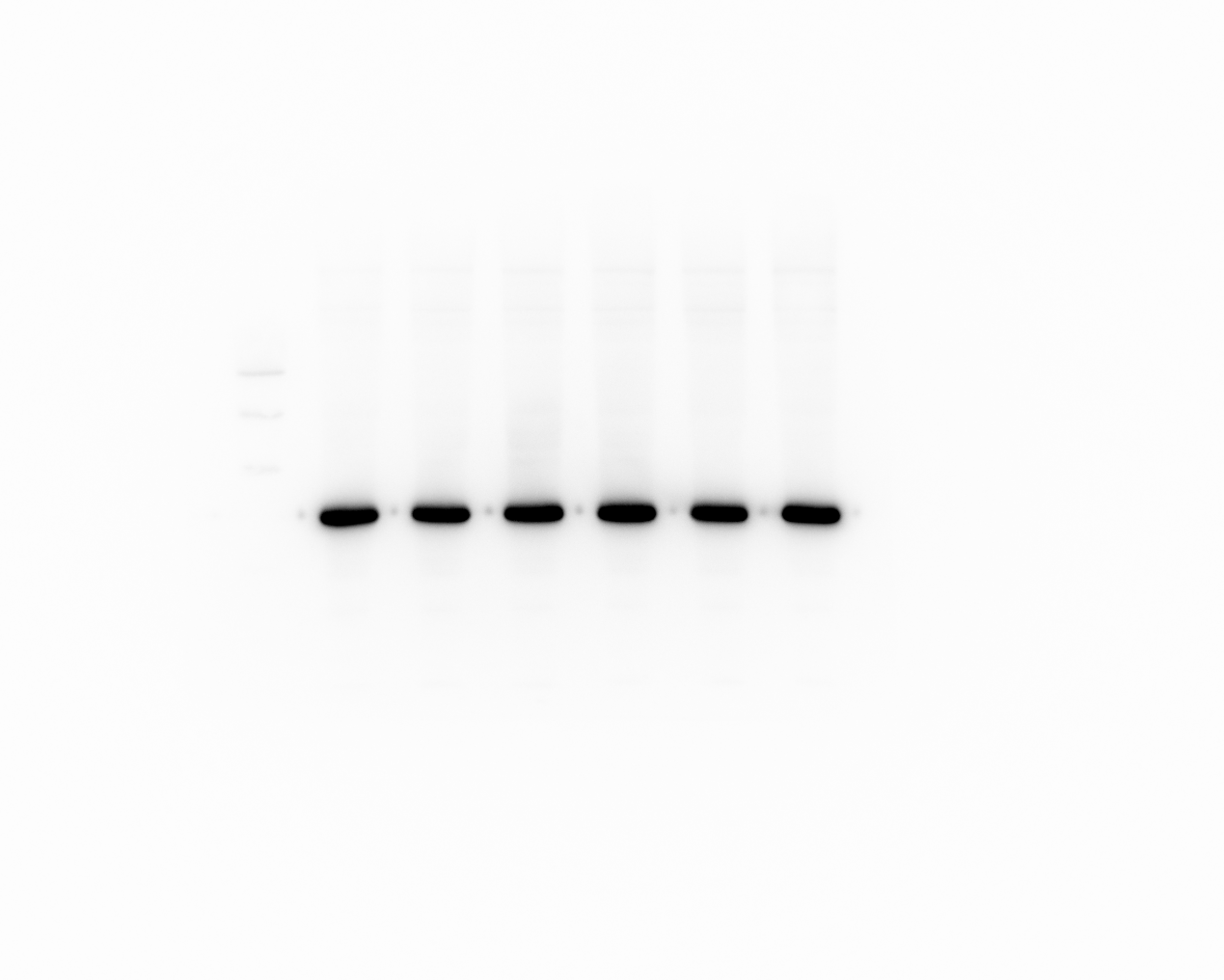


Groups (From left to right)：(1) Sham; (2) OVX; (3) OVX+ Ginkgetin (low); (4) OVX+ Ginkgetin (mid); (5) OVX+ Ginkgetin (high); (6) OVX+PGE.
