# Supplementary figures and images for: Ginkgetin attenuates bone loss in OVX mice by inhibiting the NF-κB/IκBα signaling pathway
Source: PeerJ. 2024 Jul 10;12:e17722. doi: 10.7717/peerj.17722 (PMC11246017; doi:10.7717/peerj.17722)

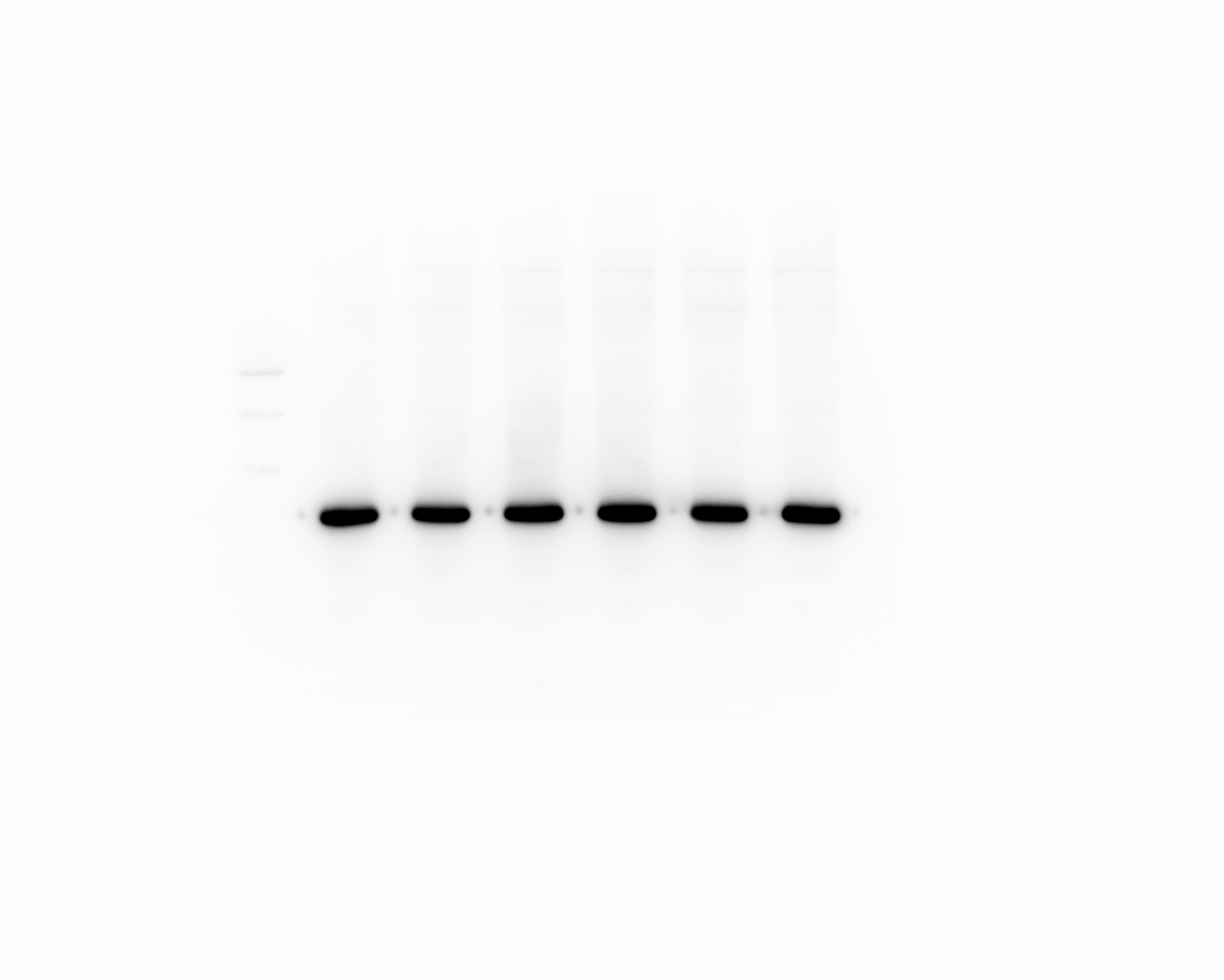

Supplement: Supplemental Information 1 [file peerj-12-17722-s001.zip › GAPDH.tif]

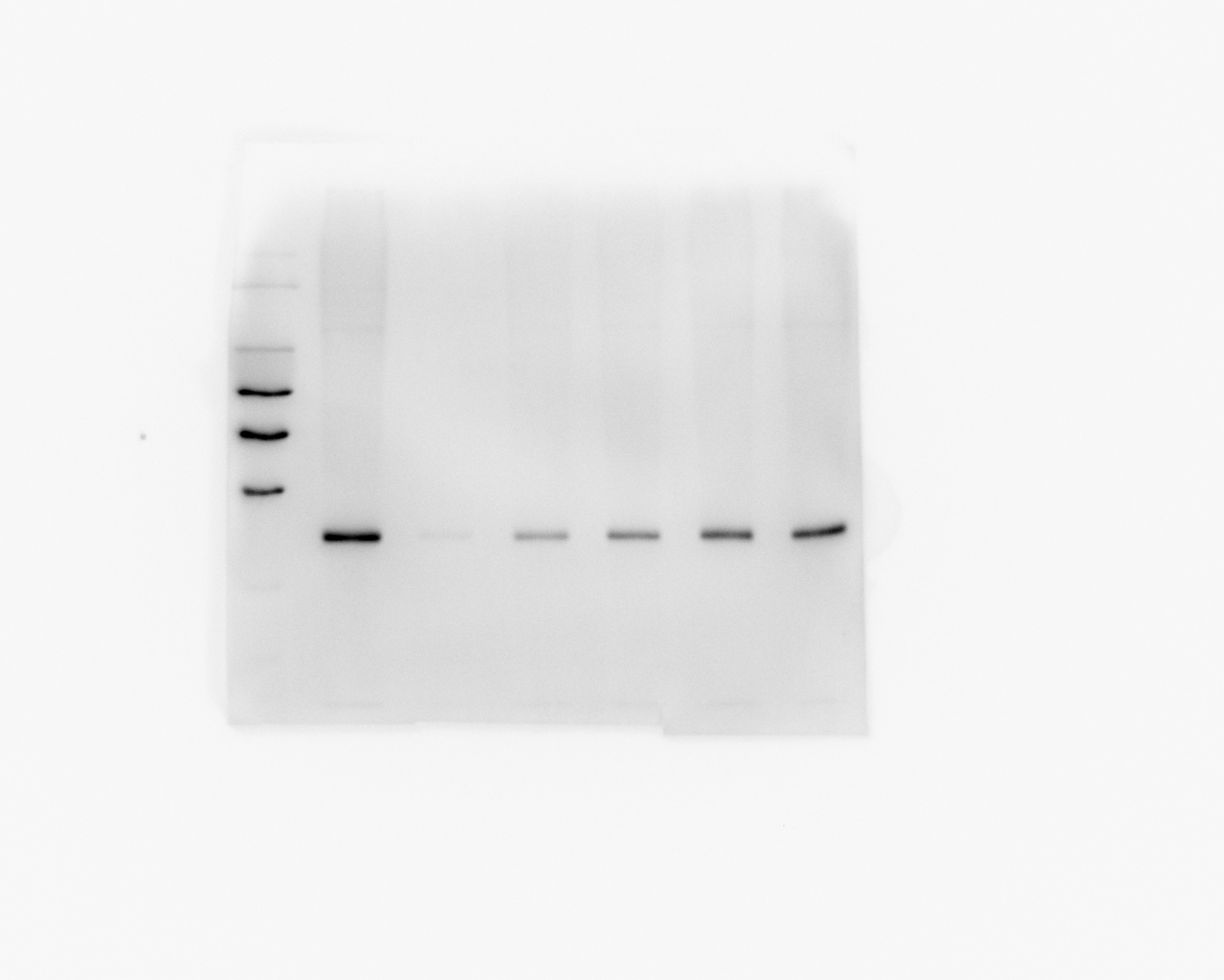

Supplement: Supplemental Information 1 [file peerj-12-17722-s001.zip › IKBa.tif]

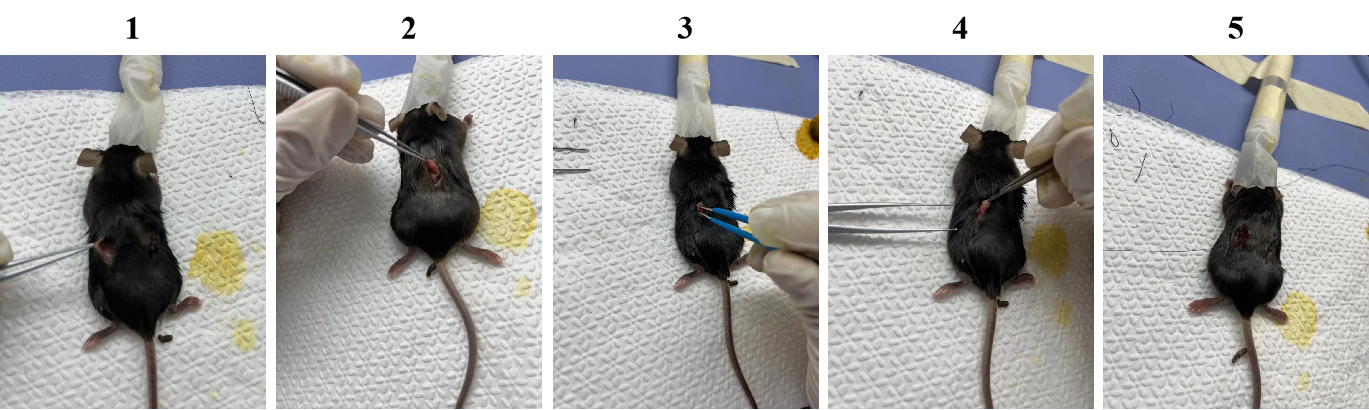

Supplement: Supplemental Information 1 [file peerj-12-17722-s001.zip › OVX.png]

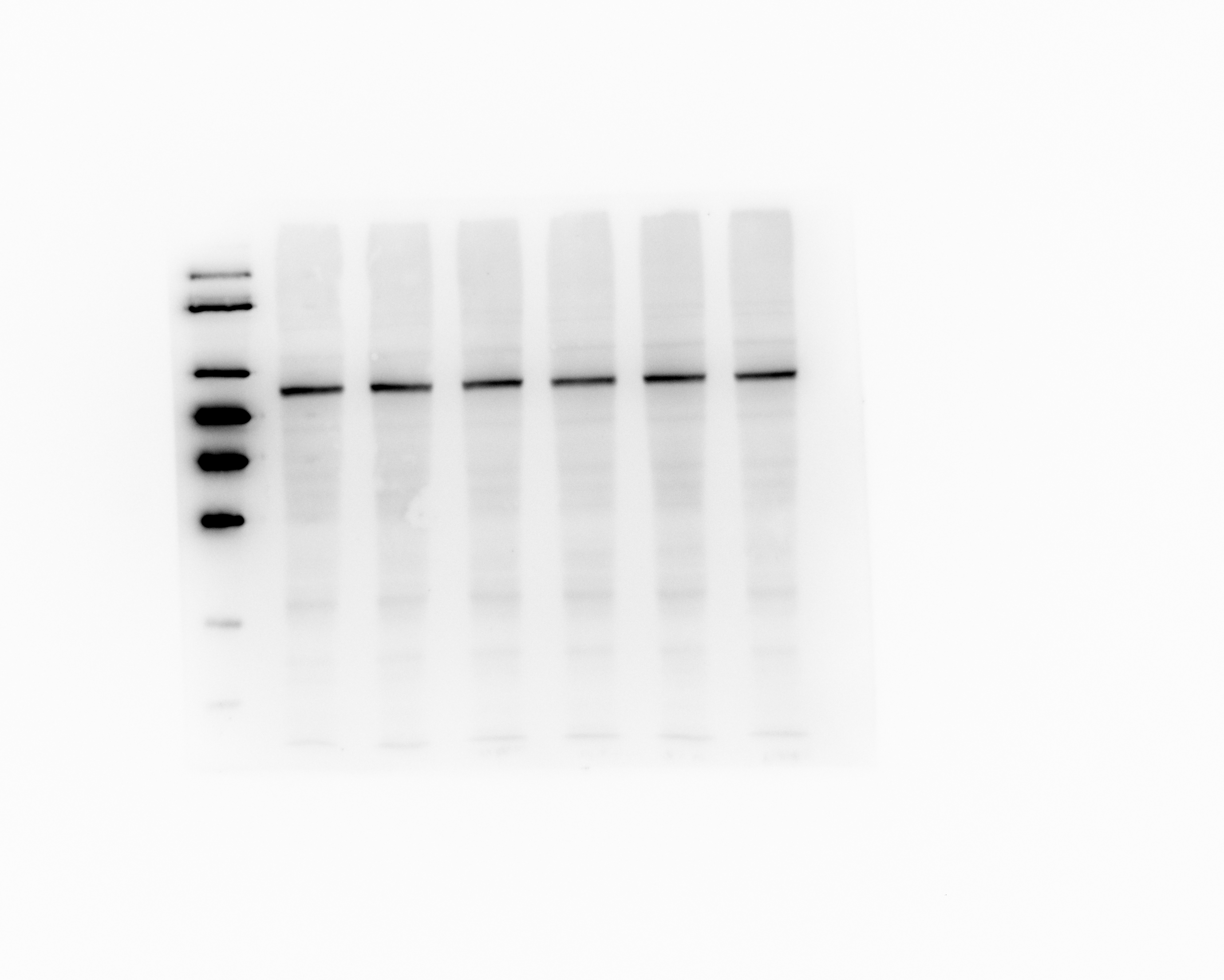

Supplement: Supplemental Information 1 [file peerj-12-17722-s001.zip › P65.tif]

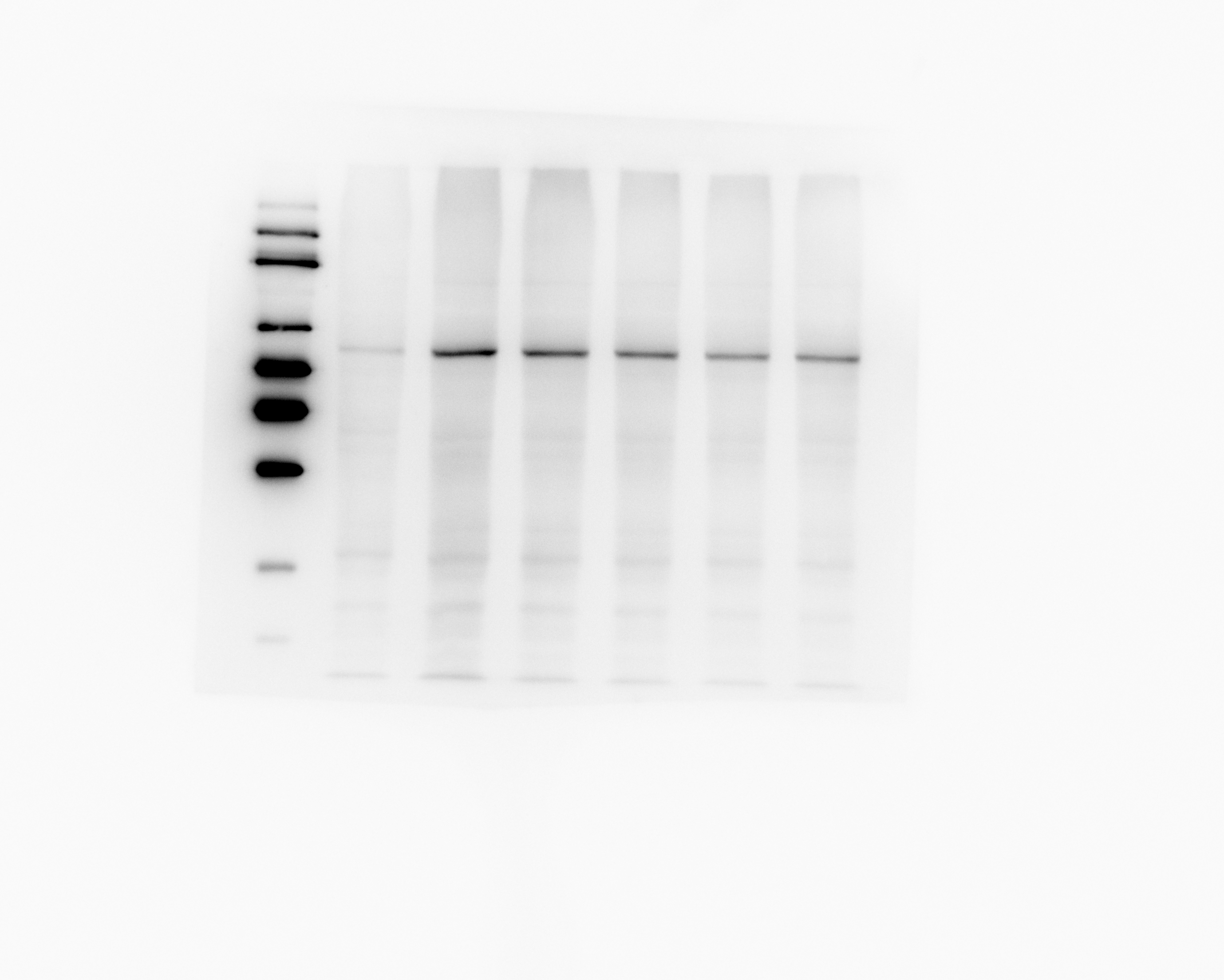

Supplement: Supplemental Information 1 [file peerj-12-17722-s001.zip › PP65.tif]

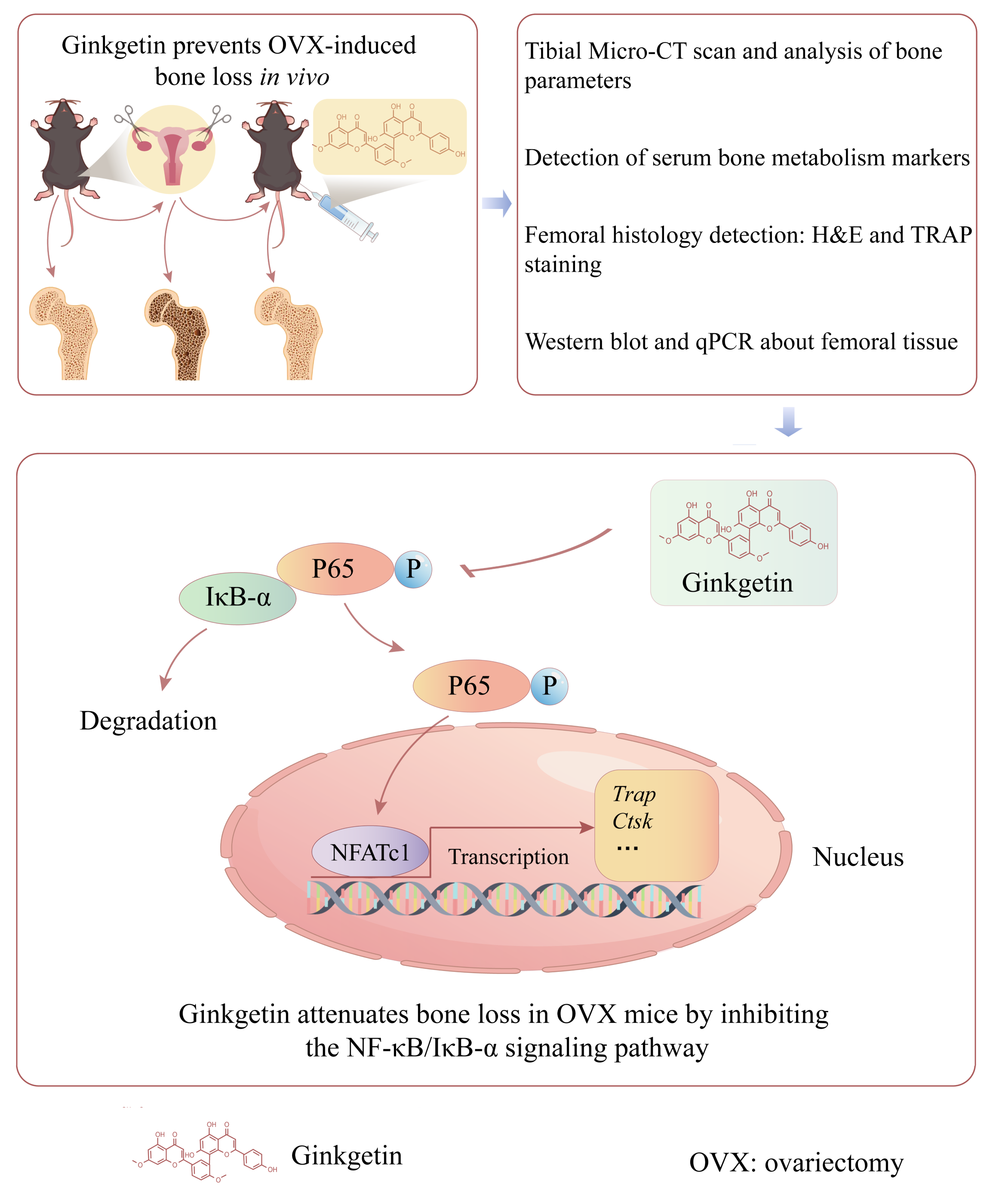

Supplement: Supplemental Information 4 — Created using Figdraw (https://www.figdraw.com/static/index.html#/) and Adobe Illustrator. [file peerj-12-17722-s004.png]
