# Supplementary material for: Ginkgetin attenuates bone loss in OVX mice by inhibiting the NF-κB/IκBα signaling pathway
Source: PeerJ. 2024 Jul 10;12:e17722. doi: 10.7717/peerj.17722 (PMC11246017; doi:10.7717/peerj.17722)
Supplement: Supplemental Information 1 [file peerj-12-17722-s001.zip › Quality Analysis Report of Ginkgo Biloba Flavonoids at 2-8 °C Plant Standard Pure Detection Conditions.doc]

| **Certificate Of Analysis** | | | |
| --- | --- | --- | --- |
| **Chinese name** | **银杏双黄酮** | **structural formula**   | |
| **English name** | **Ginkgetin** |  |  |
| **Molecular formula** | **C32H22O10** |  |  |
| **molecular mass** | **566.51** |  |  |
| **CAS** | **481-46-9** |  |  |
| **dissolve** | **dimethylformamide （DMF）** |  |  |
| **package** | **Brown sealed bottle** |  |  |
| **Loss on drying** | **≤2%** |  |  |
| **Testing items and results** | | | **notes** |
| **character** | **Yellow Powder** | **Suit** |  |
| **purity testing** | **TLC spots are clear and single, with no traces at the origin** | **Suit** |  |
| **Liquid chromatography** | **HPLC (area normalization method)≥98%** | **98.8%** | **Attached liquid phase diagram** |
| **Precautions for use** | | | |
| **Scope of use** | **This product is for laboratory scientific research only and must not be used on the human body.** | | |
| **Usage** | **After the product is restored to room temperature, take a sample in a dry environment and store it in a sealed container after sampling. Sample ready to use.** | | |
| **Storage** | **Long term storage at 2-8 ℃, sealed, stored in a cool and dry place, avoiding direct exposure to strong light.** | | |
| **Conclusion: The product quality meets the standards and is allowed to be released from the warehouse.** | | | |
| **Notes:**  **After receiving this product, users should immediately verify the variety, quantity, packaging, etc. If there are any quality or quantity issues, please contact us within 18 days from the date of receiving the product. The relevant compensation is limited to the product itself and does not involve any other losses. Thank you for your purchase.** | | | |
| **opeor： 刘丽 Q.C manager： 周红** | | | |

**Chromatographic conditions**: Determine using high-performance liquid chromatography with octadecylsilane bonded silica gel as the filler;

**Mobile phase:** methanol: 0.5% acetic acid aqueous solution (85:15);

**Flow rate:** 1.0mL/min;

**Detection wavelength:** 330nm

**Attachment:** HPLC chromatogram
